# Identifying Potential Factors Associated with High HIV viral load in KwaZulu-Natal, South Africa using Multiple Correspondence Analysis and Random Forest Analysis

**DOI:** 10.1186/s12874-022-01625-6

**Published:** 2022-06-17

**Authors:** Adenike O. Soogun, Ayesha B. M. Kharsany, Temesgen Zewotir, Delia North, Ropo Ebenezer Ogunsakin

**Affiliations:** 1 School of Mathematics, Statistics and Computer Science, College of Agriculture Engineering and Science, University of KwaZulu-Natal, Westville Campus, Durban, South Africa; 2 Centre for the AIDS Programme of Research in South Africa (CAPRISA), University of KwaZulu-Natal, Durban, South Africa; 3 Biostatistics Unit, Discipline of Public Health Medicine, School of Nursing & Public Health, College of Health Sciences, University of KwaZulu-Natal, Durban, South Africa

**Keywords:** HIV RNA viral load, Multivariate analysis, Dimensionality reduction, Burt matrix, Inertia, Random Forest, Multiple correspondence analysis, South Africa

## Abstract

**Background:**

Sustainable Human Immunodeficiency Virus (HIV) virological suppression is crucial to achieving the Joint United Nations Programme of HIV/AIDS (UNAIDS) 95–95-95 treatment targets to reduce the risk of onward HIV transmission. Exploratory data analysis is an integral part of statistical analysis which aids variable selection from complex survey data for further confirmatory analysis.

**Methods:**

In this study, we divulge participants’ epidemiological and biological factors with high HIV RNA viral load (HHVL) from an HIV Incidence Provincial Surveillance System (HIPSS) sequential cross-sectional survey between 2014 and 2015 KwaZulu-Natal, South Africa. Using multiple correspondence analysis (MCA) and random forest analysis (RFA), we analyzed the linkage between socio-demographic, behavioral, psycho-social, and biological factors associated with HHVL, defined as ≥400 copies per m/L.

**Results:**

Out of 3956 in 2014 and 3868 in 2015, 50.1% and 41% of participants, respectively, had HHVL. MCA and RFA revealed that knowledge of HIV status, ART use, ARV dosage, current CD4 cell count, perceived risk of contracting HIV, number of lifetime HIV tests, number of lifetime sex partners, and ever diagnosed with TB were consistent potential factors identified to be associated with high HIV viral load in the 2014 and 2015 surveys. Based on MCA findings, diverse categories of variables identified with HHVL were, did not know HIV status, not on ART, on multiple dosages of ARV, with less likely perceived risk of contracting HIV and having two or more lifetime sexual partners.

**Conclusion:**

The high proportion of individuals with HHVL suggests that the UNAIDS 95–95-95 goal of HIV viral suppression is less likely to be achieved. Based on performance and visualization evaluation, MCA was selected as the best and essential exploration tool for identifying and understanding categorical variables’ significant associations and interactions to enhance individual epidemiological understanding of high HIV viral load. When faced with complex survey data and challenges of variables selection in research, exploratory data analysis with robust graphical visualization and reliability that can reveal divers’ structures should be considered.

**Supplementary Information:**

The online version contains supplementary material available at 10.1186/s12874-022-01625-6.

## Background

In 2020, globally, 36 million adults over the age of 15 were living with HIV [1], South Africa contributes approximately 22% of the worldwide HIV burden, with a projected 7·8 million South Africans living with HIV and KwaZulu-Natal province being the epicentre [2]. The Fifth South African National HIV Prevalence, Incidence, Behaviour, and Communication Survey (SABSSM V) showed that of adults living with HIV, 84.8% were aware of their HIV positive status, of whom 70.7% were presently on ART, with 87.4% of these appraised to have suppressed viral load as at the end of 2019 [2]. Globally, of people living with HIV, 84% knew their status, 73% were accessing treatment, and 66% were virally suppressed in 2020 [1].

To address the constant high HIV prevalence and fast-track the response to HIV and AIDS, the South African Government implemented the Joint United Nations Programme on HIV/AIDS (UNAIDS) 95–95-95 linkage to care and treatment targets towards achieving the end of the AIDS epidemic by the year 2030 [3]. The first 95 aimed at 95% of the people living with HIV to know their HIV-positive status, whilst the second 95 aimed at 95% of all people with diagnosed HIV infection, to be obtaining sustained antiretroviral therapy (ART), while the third 95 aimed at 95% of all people obtaining ART to be attaining HIV viral suppression [3]. These targets translate mathematically towards ensuring that 86% of all people living with HIV are virally suppressed to generate profound health and economic benefits further; and achieve the sustainable goals towards HIV epidemic control, [3] which aim to end the AIDS epidemic by the year 2030.

In 2013, the World Health Organization (WHO) recommended viral load measurement as the gold standard to improve treatment efficacy [4]. HIV viral load represents the solitary utmost significant predictor for forwarding mediation [4, 5], and the lesser the viral load, the lesser the viral mediation likelihood [4–8]. In the case of Africa, South Africa’s response to HIV and AIDS has evolved rapidly over the last few years through the setting of ART treatment and guidelines in an integrated health system with holistic patient focus by setting strategic goals and standards. The country’s primary goal for ART delivery is to decrease HIV-related morbidity and mortality, achieve sustainable HIV viral suppression, and reduce the HIV transmission potential [9–11]. Despite the ART treatment policy, new HIV infections remain high, underlying transmission dynamics within communities are not well understood, and the UNAIDS target has not been met in this community [11–13]. Thus, it is critical to identify individual-level determinants of low and high HIV viral load.

Data visualisation is an integral part of data exploratory analysis and data mining [14–16]. This strengthens the facts and give basis for conclusion on further statistical analysis. Epidemiology, public health, and medical research data often comes with complexity of variables selection for statistical modelling. Although literature review could aid in variable selection, however, divers’ statistical techniques for data exploration could solve this problem. Tools such as multi-dimensional scaling, latent class analysis, convolution (for spatial and temporal data) have been suggested for exploring, displaying, and analysing complex data [17]. Furthermore, multivariable techniques commonly used for exploratory analysis include multiple correspondence analysis (MCA), principal components analysis (PCA), and factor analysis (FA) [18, 19]. Unlike PCA and FA, which are designed for continuous variables, MCA is appropriate for categorical variables. MCA has received much attention in different fields, particularly in transportation [20–22], engineering [23], social science fields [24–26], and most importantly in health [27–32] and less so to epidemiological studies and particularly in HIV. Similarly, Random Forest analysis (RFA), a multipurpose supervised machine learning approach in applied statistics used for classification and prediction was applied [33, 34]. RFA has gained popularity with the advent use of machine learning algorithm for HIV prevention intervention [35].

This study’s objectives were to explore binary, nominal, and ordinal variables from a large complex dataset. MCA and RFA were used to conduct an in-depth review, explore patterns, and identify potential factors contributing to  HHVL, with the aim of a minimal loss of information.

## Methods and material

### Study area and population

This study analyzed data from the comprehensive HIV Incidence Provincial Surveillance System (HIPSS), conducted in rural Vulindlela and the peri urban Greater Edendale area in the Msunduzi municipality uMgungundlovu district of KwaZulu-Natal Province in South Africa. The study aimed to monitor HIV-related measures and assess the association of the contemporaneous programmatic scale of HIV prevention and treatment efforts in a “real world,” non-trial setting on HIV prevalence and incidence [11, 12]. Two sequential cross-sectional surveys were undertaken from 11 June 2014 to 18 June 2015 (2014 Survey) and 8 July 2015 to 7 June 2016 (2015 Survey). Furthermore, the rationale, design, objectives, and methods of HIPSS have been fully described in past studies [11–13]. 

### Study procedures

Following written informed consent, a face-to-face questionnaire was administered to collect demographics, socio-economic status, and health-related information. To minimize misclassification, all participants had HIV-antibody and viral load testing using multiple assays with high sensitivity and specificity. The study procedures have been described elsewhere [10, 11]. Level of ARV drugs were measured in the plasma sample of participants to assess the accuracy of self-reported ARV drug use.

### Study design and data

Households were randomly selected using two-stage random sampling methods, and one individual per household, within the age range 15–49 years, was randomly selected and enrolled. In the 2014 and 2015 Surveys, 9812 and 10,236 individuals were enrolled. Following participants’ HIV antibody and viral load testing, a total of 3969 (2014 survey) and 3870 (2015 survey) tested positive for HIV. However, only 3956 and 3868 had viral load measurements, with missing data for  11 and 2 participants in the survey period 2014 and 2015, respectively. This paper used the data of those diagnosed with HIV and having their viral load measurement. The primary outcome of this paper is viral load profile which we categorized as HIV viremia of ≥400 copies/ml (high HIV RNA viral load (HHVL)) and < 400 copies/ml (low HIV RNA viral load (LHVL)) among individuals living with HIV at the population level [9, 10, 36, 37].

### Statistical analysis

A descriptive analysis was performed  using SAS (SAS Institute, Cary, North Carolina) version 9.4 to characterize the sample; weighted percentage accounted for the sample weights. The Open Bug statistical software R (version 4.0.2), using packages “FactoMineR” and “factorextra” was used for the MCA analysis, while packages “randomforest” and “caret” were used for RFA. Further, we conducted MCA to assess any form of associations between levels of indicators. This approach is a data mining technique that allows researchers to analyze a critical categorical data set with several indicators and position response categories [18, 38, 39]. For this approach to be executed, the initial stage required restructuring of the data matrix, and the variables of interest in columns (socio-demographic, behavioral, psycho-social, and biological variables), variables and categories names were recoded to abbreviate their names for easy reading on MCA maps (see Additional file 3: Table S3). One of the benefits of this approach is the ability to cluster the various relevant levels of indicators through diagrammatic means together. Additionally, the discrimination measures (DM) are further pertinent to individual dimension creation, and the centroid coordinates assist in distinguishing individual classes on the visceral map. In addition, the RFA method was deployed to classify high-rank predictors linked with HHVL. The variable of interest was HIV viral load status which was defined as HIV viremia of ≥400 copies/ml (HHVL) and < 400 copies/ml (LHVL) among people living with HIV (PLHIV) at the population level. This cut-off for HIV viral load was applied as several studies have shown the less likely potential for HIV viral transmission at this value [37, 40].

### Multiple correspondence analysis

As a data mining approach, MCA is a multi-component addendum of correspondence analysis (CA) that shows patterns and associations between various indicators [41, 42]. Besides, it can also show the intricate fundamental patterns links between indicators in the absence of formulating hypotheses [43, 44].

### Specification of MCA

Considering a situation in which a row by column matrix is made on an, *I* × *J* indicator matrix that comprises conventional *i* individual responses, HHVL, and *J* is the set of levels of contributing factors. Based on this specification, the constituent in the cell (i, j) entails the individual responses *i* and class *j* [43]. The endpoint of this formulation yielded MCA plots, and the linked groups are positioned to individual. These contributions of the rows and columns help locate the observations or variables that are of importance to a given factor. Similarly, two distinct cloud points are formed from MCA for indicators classes, and individual variables are characterized on a two-dimensional chart. Consequently, this cloud relies on the individual distance between indicators having diverse groups. Below is the mathematical representation

i $${d}_q^2\left(i,i\hbox{'}\right)=\frac{1}{f_k}+\frac{1}{f_{k\hbox{'}}}$$

In this case, *f*_*k*_ and *f*_*k*'_ are the comparative rate of individual responses designated group in *k* and *k*' respectively. Furthermore, the overall squared distance between two individual responses is obtained by totalling all individual square distances, as given in eq. (2)

ii $${D}^2\left(i\hbox{'},i\hbox{'}\right)=\frac{1}{N}\sum \limits_{n\varepsilon N}{d}_n^2\left(i,i\hbox{'}\right)$$

Where *D*^2^(*i*', *i*') denote the total squared distance between individuals *i* and *i ‘* and ${d}_n^2$ (*i*, *i*') represent the squared distance between individuals *i* and *i ‘* for variable *n*, such that *N* implies the set of all variables. Also, the squared distance between categories *k* and *k*' is represented in equation (iii).

iii $${\left(K,K\hbox{'}\right)}^2=\frac{n_k+{n}_{k\hbox{'}}-2{n}_{kk\hbox{'}}}{{n}_k{n}_{k\hbox{'}}\left/ n\right.}$$

Where (*K*, *K*')^2^ represent the squared distance between categories *k* and *k*', *n*_*k*_ and *n*_*k*'_ represent the number of individuals that designated group *k* and k ‘ respectively, while n and *n*_*kk*'_ connotes to the total amount of individual responses and amount of individuals that designated levels *k* and *k*' respectively. Based on previous studies, *n*_*kk*'_ it will tend to zero when levels *k* and *k*' are two levels of similar indicator [45, 46].

As Greenacre (1984) proposed, the calculation of the elucidated inertia for individual dimensions in MCA frequently undervalues the superiority of fit, thereby suggesting the need for an adjustment to the computation using the Burt matrix as against the indicator matrix [47]. Recently, Greenacre and Blasius’ (2017) work strengthened and remedied the proportion of inertia in MCA by merely using a scale readjustment of the MCA [43]. Therefore, borrowing from the strength of this technique, total inertia is sedate by averaging inertia of all off-diagonal blocks of *C*, by confiscating the fixed contributions of the diagonal blocks. In the mathematical expression, below is the representation:

$$average\ off- diagonal\ inertia\kern0.5em =\frac{Q}{Q-1\ }\ \left( inertia\ (C)-\frac{J-Q}{Q^2}\right)$$

Assume part of the inertia is calculated from the principal inertia ${\lambda}_s^2$ of $C\ or\ from\ the\ principal\ inertia\ s\ {\lambda}_s of\kern0.5em I; hence\ for\ each\ {\lambda}_s^2\ge 1 \left/ Q,\right.$ the adjusted inertias are calculated as follows:

$${\lambda}_s^{adj}={\left(\frac{Q}{Q-1}\right)}^2\ {\left({\lambda}_s\kern0.5em -\frac{1}{Q}\right)}^2$$

As a result of optimality scale values’ attractive properties, adopting MCA using Burt matrix are most recommended and used in this paper’s final interpretation.

### Random forest analysis

Random forest analysis (RFA) is a multipurpose supervised algorithm used for classification or regression [33, 34]. Is used for variable screening and dimension reduction in high dimensional data set where covariate selection and ranking are very important for prediction and interpretation. RFA is gaining popularity in many fields of health sciences due to its ability to detect high estimate precision and outputs information on the relevance of indicators for the classification problem in our data [48]. RFA algorithm outputs the importance of various predictor variables for outcome of interest. In RFA, the out-of-bag (OOB) error rate for classification can be obtained through the built-in-cross-validation algorithm, in which data are grouped into training (bootstrap) and test data [34, 48]. The data was further calibrated through the bagging process by randomly selecting samples into training (70%) and testing (30%) dataset to assess their performance due to its strong predictive capability. Summarily, randomness in RFA algorithm can reduce overfitting by (i) building multiple trees; (iii) portrays observations with replacements; and (iii) splitting nodes on the best split within a random subset [33]. Recent study ascertained the accuracy of RFA against other analytical tools for data exploration and classification [49]. In our study, RFA was used to assess the relative importance of the explanatory variables in classifying the viral load profile of HIV positive individual. In assessing the success of a methodology, accuracy is an essential criterion. Divers’ metrics are used for evaluating importance or significant variables, without a universal standard of assessment, multiple techniques are often used. Suitably, the approach outputs the standing of the various indicators for dependent variables through Mean Decreases Accuracy (MDA) and Mean Decrease Gini (MDG). These indicators are referred to and calculated as mean decrease in accuracy divided by estimated standard error. Which is the standardized measure of identifying high important predictive variables in RFA [49, 50]. The highest decrease in the accuracy and Gini values of the model implies the best predictive and the most important variable respectively [49]. The uppermost reduction in the precision of the model denotes the superlative predictive and furthermost relevant indicator, respectively [49]. This study used statistical measure such as high importance predictors plot and mean score to assess important factors associated with HHVL. Hence, both MCA and RFA were used to evaluate the most important predictors associated with HHVL and to examine relationship and patterns among several categorical variables in HIPSS data.

## Results

### Descriptive statistics of study participants

The present study has utilized the responses drawn from the 3956 (2014 Survey) and 3868 (2015 Survey) HIV-positive sample. From Additional file 1 (Supplementary Table S1), the descriptive statistics of participants shows that high viral load rate decreased by 9.1%, from 50.1% in 2014 to 41.0% in 2015. Across both surveys, over 70% of the participants were women, with more than half having incomplete high school education (53.2%, 59.5%). In comparison, the majority (84.5%, 81.1%) were never married, more than half (54.9%, 55.2%) have an income of ZAR ≥2500, (60.4%,26,9%) were accessing health care in 2014 and 2015 respectively. The majority, 76.1% (87.6%), were sexually active in the last 12 months, while 84.2% (83.3%) had two or more sex partners in their lifetime. HIV knowledge and testing history show that the majority, 55.9% (68.5%), had two or more HIV tests in their lifetime, while few 25.1% (14.92%) had the perception that they are not likely to contract HIV, knowledge of HIV status was high 58.9% (72.2%) but far from the USAID target. In 2014(2015), respectively, 43.4% (57.5%) were on ART; 79.7% (88%) were on fixed dose, with almost half 48.5% (53.6%) of those living with HIV having a CD4 cell count of ≥500 cells per μL.

**Fig. 1:**
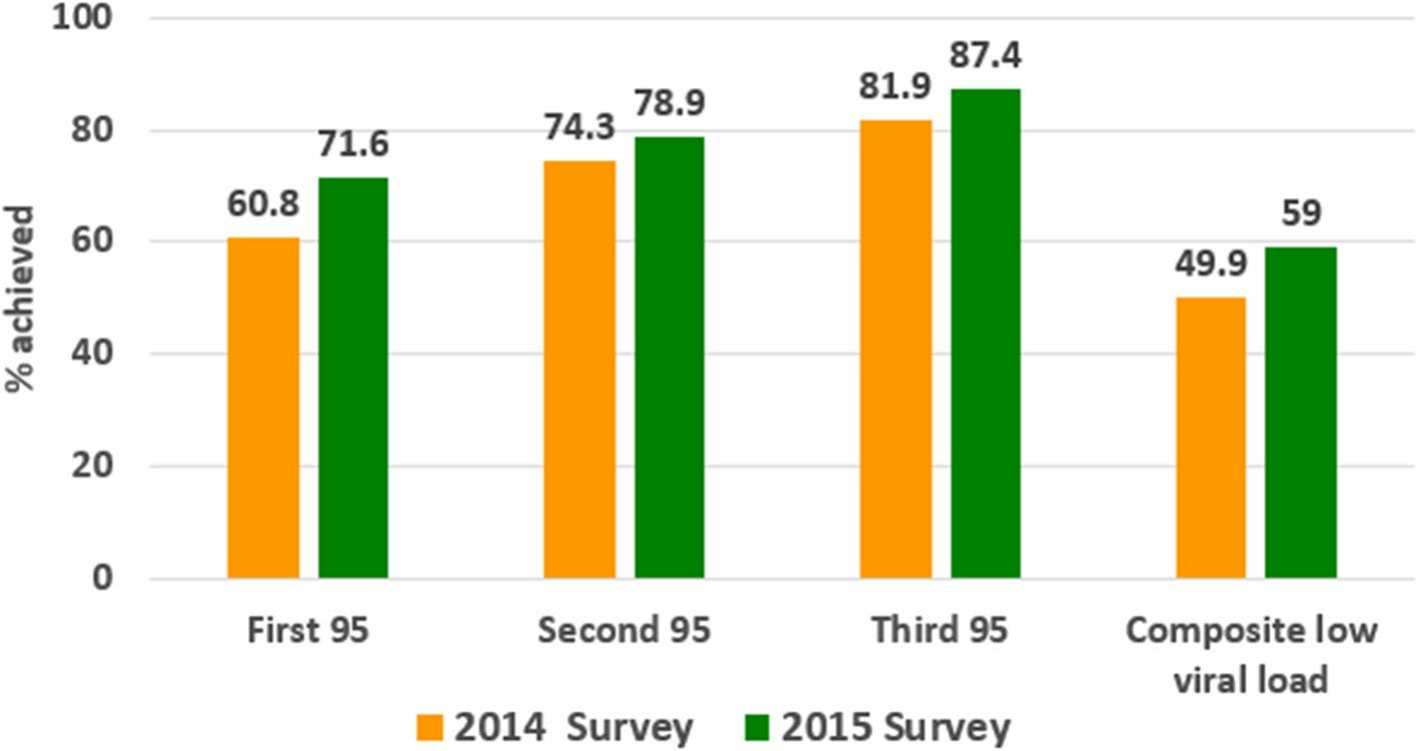
Progress towards 95–95-95 targets of participants (2014–2015) . First 95: percentage of PLHIV who are aware of their HIV status; Second 95: percentage of those who knew their status and on ART; Third 95: percentage of those on ART and with low viral load; Composite low viral load: percentage of PLHIV with low viral load

As shown in Fig. 1, among all HIV positive men and women, overall composite LHVL was 49.9% (95% CI: 47.3–52.7) in 2014 and  increased to 59.0% (95% CI 56.0–62.0) in 2015 survey. Progress towards UNAIDS 95–95-95 target shows that among people living with HIV 60.8% (95% CI 58.4–63.4) knew their HIV status, of which 74.3% (95% CI 71.8–76.8) had initiated ART, from those on ART, 81.9% (95% CI 79.3–84.4) have attained a low viral load of < 400 copies/ml in 2014 survey. While in 2015 survey, 71.6% (95% CI 69.9–73); 78.9% (95% CI 77.1–80.7); and 87.4% (95% CI 85.8–89.0) were attained. (See Additional file 1: Table S2 for absolute number of each element).

### MCA results

Findings from the MCA using the Burt matrix are presented in Table 1. For both years, the first dimension accounted for 31.36% (32.97%), while the second dimension accounted for 12.31% (9.58%) of the total variance in the year 2014 (2015), respectively. The total cumulative variance for dimensions 1 and 2 is 43.68% for the 2014 Survey and 42.55% for the 2015 Survey data. This shows a considerably higher degree of data variability. Similarly, for each dimension, the magnitude of information is determined by the eigenvalues (also known as inertia), which take on values between 0 and 1. A lower eigenvalue confirms that variables are heterogeneous. A scree plot is a standard method used in assessing the most appropriate number of depth and the proportions of variance described [41, 42]. Fig. S1 in Additional File 2 displays the scree plot, which aids in the visualization of the proportion of variance explained and drops faster and significantly from dimensions 1 to 2. This further confirms that the first two dimensions are suitable to explore the study variables further. The diagrammatic depiction of MCA has aided in interpreting the associations among high HIV viral load parameters.

**Table 1:** Greenacre adjustment to inertia of eigenvalues and variances of the top ten dimensions

Dimension	2014 Survey			2015 Survey		
	Eigenvalue	% of variance	Cumulative % of variance	Eigenvalue	% of variance	Cumulative % of variance
1	0.04	31.36	31.36	0.04	32.97	32.97
2	0.01	12.31	43.68	0.01	9.58	42.55
3	0.01	6.05	49.73	0.00	6.16	48.71
4	0.01	5.30	55.02	0.00	4.31	53.02
5	0.01	4.02	59.05	0.00	3.93	56.95
6	0.00	3.30	62.34	0.00	3.72	60.68
7	0.00	2.93	65.27	0.00	2.63	63.30
8	0.00	2.48	67.75	0.00	2.26	65.53
9	0.00	2.31	70.07	0.00	2.02	67.55
10	0.00	1.87	71.94	0.00	1.91	67.46

**Fig. 2:**
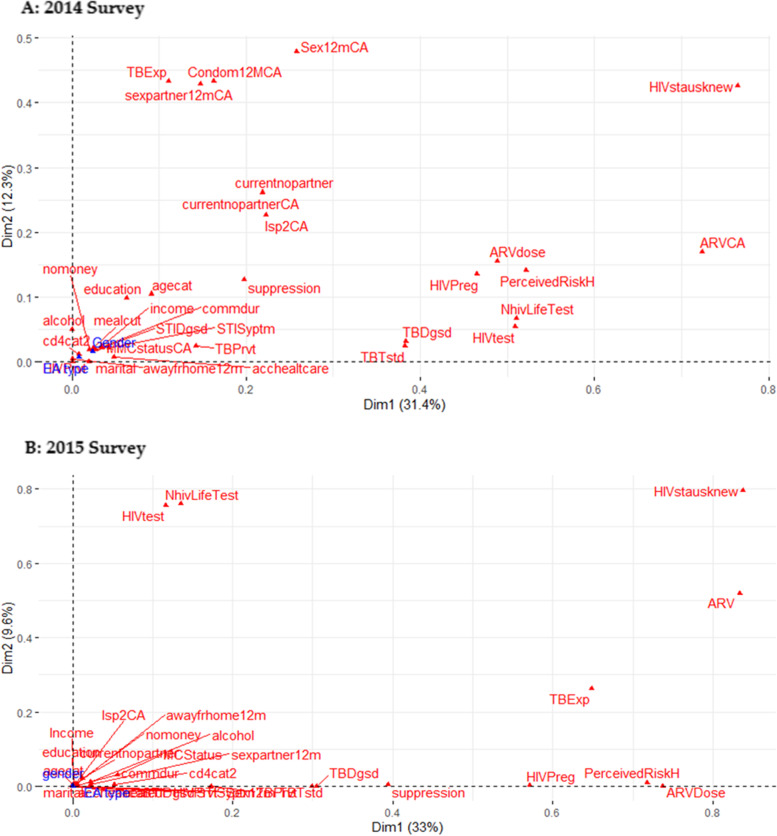
Multiple correspondence analysis plot showing association between variables and patterns (**A**) 2014 and (**B**) 2015 survey. Factors contributing to high viral load (≥ 400 copies m/L) in A = 2014 survey were: HIVstatusknew, ARV, ARVdose, perceivedRiskH, NhivLifeTest, HIVtest, HIVPreg, TBDgsd, TBTstd, Sex12mCA, condom12MCA, TBExp, sexpartner12mCA, currentnopartner, and lsp2CA. while B = 2015 survey were: HIVstatusknew, ARV, TBExp, ARVDose, PerceivedRiskH, NhivLifetest and HIVtest . As denoted by distance of variable away from  centre point. See Table S3 in Additional file 1 for all variables and categories full descriptions

The most and less contributing factors and associations between variables and patterns across the 2014 Survey and 2015 Survey in the first two dimensions are shown in Fig. 2. In the 2014 survey (Fig. 2a), most predictors variables identified are knowledge of HIV status, being on ART, ART dosage, perceived risk of contracting HIV, number of lifetime HIV test, sex partner in the last 12 months, current sext partner, lifetime sex partner, exposed to tuberculosis in the last 12 months and ever diagnosed of TB. While in 2015 (Fig. 2b) also shows knowledge of HIV status, being on ARV, ARV dosage, number of lifetime HIV tests. Similarly, fewer contributing factors were found to be all variables that were closer to the center of the map. This plot also revealed possible variables with multicollinearity; in the 2014 survey, these are, number of current sex partners and lifetime sex partners; perceived risk of contracting HIV, number of lifetime HIV test and ARV dosage; Exposed to TB last 12 months and number of sex partner last 12 months.

**Fig. 3:**
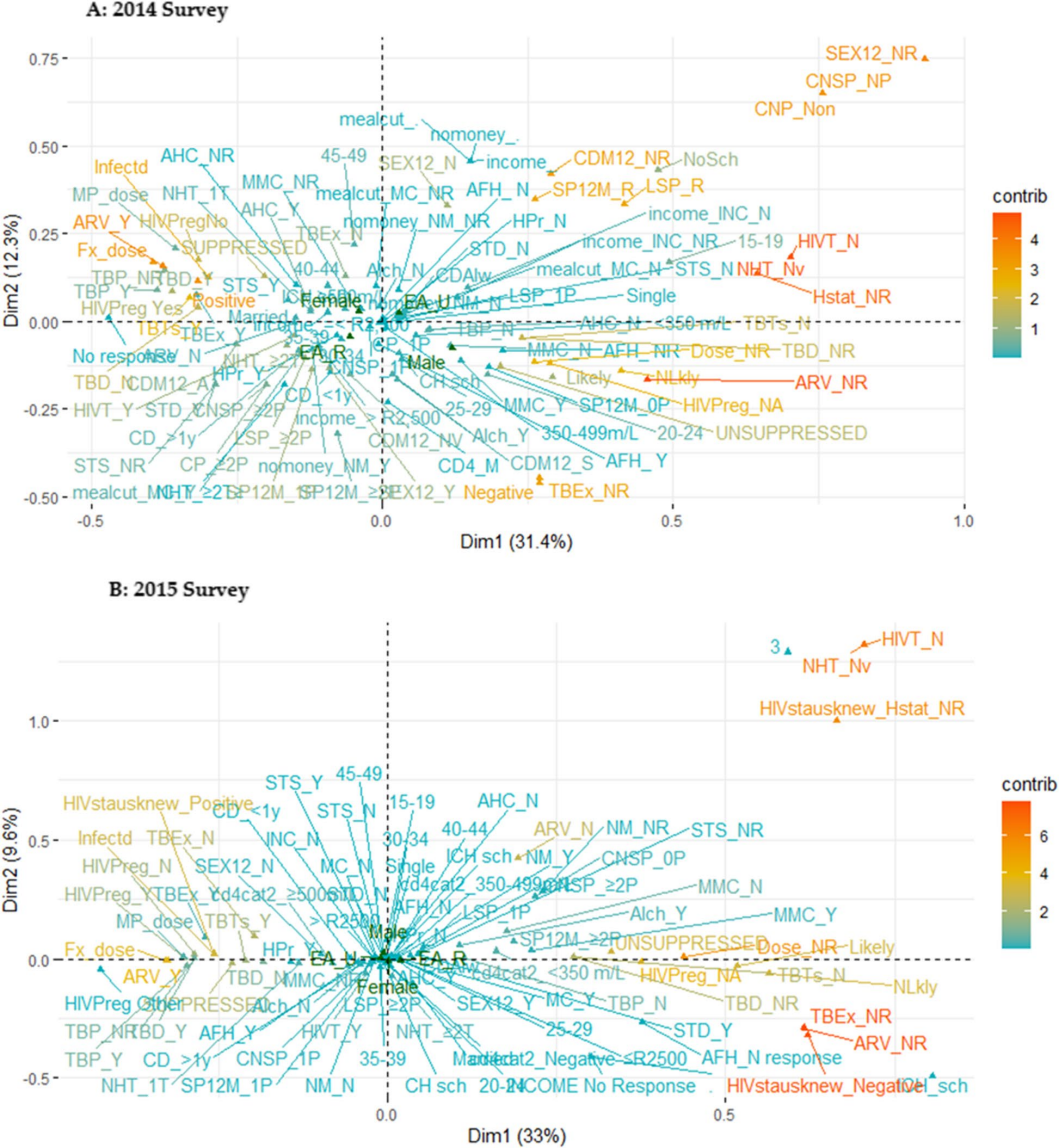
Multiple correspondence analysis plot showing contribution of variables categories to high viral load (≥ 400 copies m/L). (A) 2014 and (B)2015 survey. Variable categories in red contributed most followed by those in orange color, these are: **A** = 2014 survey: ARV_N, Hstat_NR, NHT_Nv, HVT_N, CNP_Non, SEX12_NR, CDM12_NR, SP12M_NR, LSP_NR, **B** = 2015 survey: ARV_N, HIVstat_negative, HIVsta_NR, TBEx_NR, HVT_N, NHT_Nv . See Table S3 in Additional file 1 for these categories’ full description

The graphical plots in Fig. 3 illustrate the total depiction of groupings with the maximum contributions in the individual quadrant in the 2-dimensional plot. We noted that those variables categories linked to high HIV viral load in 2014 were located at the bottom right quadrant of our plot. While in 2015, it was located at the right quadrant of the plot. Variable categories in red contributed most, followed by those in orange color**.** In 2014 (Fig. 3a), most contributing variables categories were ARV_NR, Hstat_NR, HIVT_N, NHT_Nv, (in red) followed by SEX12_NR, CNSP_NP, Self-reported HIV status: Negative, TBEx_NR, SP12M_R, (ARV_Y, Fx_Dose ARV dose_Fx, TBTs_Y, SEX12M_N, Nlkly, Dose_NR (in orange). While in 2015 (Fig. 2b), the categories of the most contributing variable are ARV_NR, TBEx_NR, HIVstatusknew_negative (in red), followed by NHT_Nv, HIVT_N, HIVstatknew_NR, ARV_N, Dose_NR, HIVPreg_NA, Perceived risk: (all categories), TBEx_N, HIVstatusknew_ positive Fx_dose, ARV_Y (in orange), see Additional File 1 supplementary Table S2 for the description of these categories. A bar chart of the same plot by each dimension was shown in Fig. S2: Additional File 2.

Furthermore, the magnitude of connotation between these variable groupings and their axis represented the square cosine (cos2) plot in Additional File 2: Fig. S3. A well-represented variable category by the two dimensions will show a cos2 close to 1, with high cos2 in red, mid cos2 in orange, and low in blue. This further confirms the strength and quality of the contributing factors identified in Fig. 2 and the relative association of variables categories in Fig. S4 in Additional File 2.

Additionally, Fig. S5 in Additional File 2 reveals the MCA biplot. MCA biplot quantifies the categories and corresponding individuals by locating their proximity if many choose the same two categories. Therefore, this MCA biplot visually identifies individuals with similar categories choice. The plot reveals a total behavioral shape in the HIV seropositive dataset such that blue themes and columns designate rows (individual participants) by red triangles. Row themes with an analogous outline are closed on the factor map and the same argument clutches for column themes. This makes available additional benefits by adding pertinent external information about visualization of individual random selection and their location proximity, which enhances spatial clustering and variation analysis.

Similarly, in Tables 2 and 3, all the indicators contributing were recognized based on the coefficient of determination  R^2^ and *p*-value. The order of presentation shows the significance of individual indicators. The  R^2^ value demonstrates the strength of the association. The value closer to zero implies no association, while a value close to one denotes an enormous association. From Tables 2 and 3, for both years, high HIV viral load was found to be strongly associated with” knowledge of HIV status,” taking ARV,” “ARV dosage, “perceived risk of contracting HIV,” “ever tested for HIV,” “number of lifetime HIV test,” and “exposure to TB in last 12 months”.

**Table 2:** Statistical significance test for variables contributing to HIV viral load unsuppression using Burt Matrix (2014 Survey)

Variable (dimension 1)	*R*^2^	*p value*	Variable (dimension 2)	*R*^2^	*p value*
Perceived Risk of contracting HIV	0.52	*<*.0001	Exposed to TB last 12 month	0.43	*<*.0001
Ever tested for TB	0.38	*<*.0001	Knowledge of HIV status	0.43	*<*.0001
Ever diagnosed of TB	0.38	*<*.0001	Number of sex partner 12 month	0.40	*<*.0001
ARV dosage	0.48	*<*.0001	Had sex in last 12 months	0.48	*<*.0001
Ever tested for HIV	0.51	*<*.0001	Condom use last 12 months	0.43	*<*.0001
Knowledge of HIV status	0.76	*<*.0001	Current number of sex partner	0.26	*<*.0001
Number of lifetime HIV test	0.51	*<*.0001	Number of lifetime sex partner	0.22	*<*.0001
Taking ARV	0.72	*<*.0001	Taking ARV	0.17	*<*.0001
Pregnant while HIV positive	0.46	*<*.0001	ARV dosage	0.15	*<*.0001
Had sex last 12 months	0.26	*<*.0001	Perceived risk of contracting HIV	0.14	*<*.0001
Number of lifetime sex partner	0.22	<.0001	Pregnant while HIV positive	0.13	*<*.0001
Number of current sex partner	0.22	<.0001	Age (in years)	0.1	*<*.0001
Condom use last 12 months	0.16	*<*.0001	Education	0.10	*<*.0001
Number of sex partners last 12 months	0.15	*<*.0001	Number of lifetime HIV test	0.07	*<*.0001
On medication to prevent TB	0.14	*<*.0001	Ever tested for HIV	0.05	*<*.0001
Exposed to TB last 12 months	0.11	*<*.0001	Alcohol consumption	0.05	*<*.0001
Age (in years)	0.09	*<*.0001	Ever Diagnosed of TB	0.03	*<*.0001
Education level	0.06	*<*.0001	Ever tested of TB	0.02	*<*.0001
Accessing health care	0.05	*<*.0001	On medication to prevent TB	0.02	*<*.0001
Had any STI symptoms	0.04	*<*.0001	Income	0.02	*<*.0001
Ever diagnosed of TB	0.03	*<*.0001	Ever diagnosed of STI	0.02	*<*.0001
Income	0.03	*<*.0001	Had STI symptoms	0.02	*<*.0001
Length of stay in community	0.03	*<*.0001	Meal cut	0.02	*<*.0001
Gender	0.02	*<*.0001	Length of stay in community	0.02	*<*.0001
Circumcision status	0.02	*<*.0001	Money loss	0.02	*<*.0001
Meal cut	0.02	*<*.0001	Gender	0.02	*<*.0001
Marital status	0.02	*<*.0001	Circumcision status	0.02	*<*.0001
Income loss	0.02	*<*.0001	Current CD4 cell counts	0.01	*<*.0001
Enumeration area	0.01	*<*.0001	Enumeration area	0.01	*<*.0001
Current CD4 cell count	0.01	*<*.0001	Accessing health care	0.01	*<*.0001
Migration history	0.01	*<*.0001

**Table 3:** Statistical significance test for variables contributing to HIV viral load unsuppression using Burt matrix (2015 Survey)

Variable (dimension 1)	*R*^2^	*p-value*	Variable (dimension 2)	*R*^2^	*p-value*
Perceived Risk of contracting HIV	0.72	*<*.0001	Taking ARV	0.52	*<*.0001
Pregnant while HIV positive	0.57	*<*.0001	Ever had HIV test	0.76	*<*.0001
Exposed to TB last 12 months	0.65	*<*.0001	Knowledge of HIV status	0.80	*<*.0001
Knowledge of HIV status	0.83	*<*.0001	Number of lifetime HIV test	0.76	*<*.0001
Taking ARV	0.83	*<*.0001	Exposed to TB in last 12 months	0.26	*<*.0001
ARV Dosage	0.73	*<*.0001	Circumcision status	0.03	*<*.0001
Ever diagnosed of TB	0.30	*<*.0001	Current number of sex partner	0.02	*<*.000
Ever tested for TB	0.30	*<*.0001	Ever tested for TB	0.30	<.0001
Alcohol consumption	0.01	*<*.0001	On medication to prevent TB	0.17	*<*.0001
Number of lifetime HIV test	0.14	*<*.0001	Perceived risk of contracting HIV	0.01	*<*.0001
Number of sex partner last 12 months	0.01	*<*.0001	Ever tested for HIV	0.12	*<*.0001
Income loss	0.01	*<*.0001	Circumcision status	0.01	*<*.0001
Number of lifetime sex partner	0.01	*<*.0001	Current CD4 cell count	0.05	*<*.0001
Gender	0.01	*<*.0001	Alcohol consumption	0.02	*<*.0001
Current CD4 cell count	0.01	*<*.0001	Number of sex partner last 12 months	0.02	*<*.0001
Age (in years)	0.01	*<*.0001	Current number of sex partner	0.01	*<*.0001
Marital status	0.00	*<*.0001	Had sex last 12 months	0.01	*<*.0001
Length of stay in community	0.00	*<*.0001	Length of stay in community	0.01	*<*.0001
On medication to prevent HIV	0.00	*<*.0001	Number of lifetime sex partner	0.00	*<*.0001
Income loss	0.00	*<*.0001			
Ever diagnosed of TB	0.00	*<*.0001			
Meal cut	0.00	*<*.0001			
Migration history	0.00	*<*.0001			
Education	0.00	*<*.0001			

**Fig. 4:**
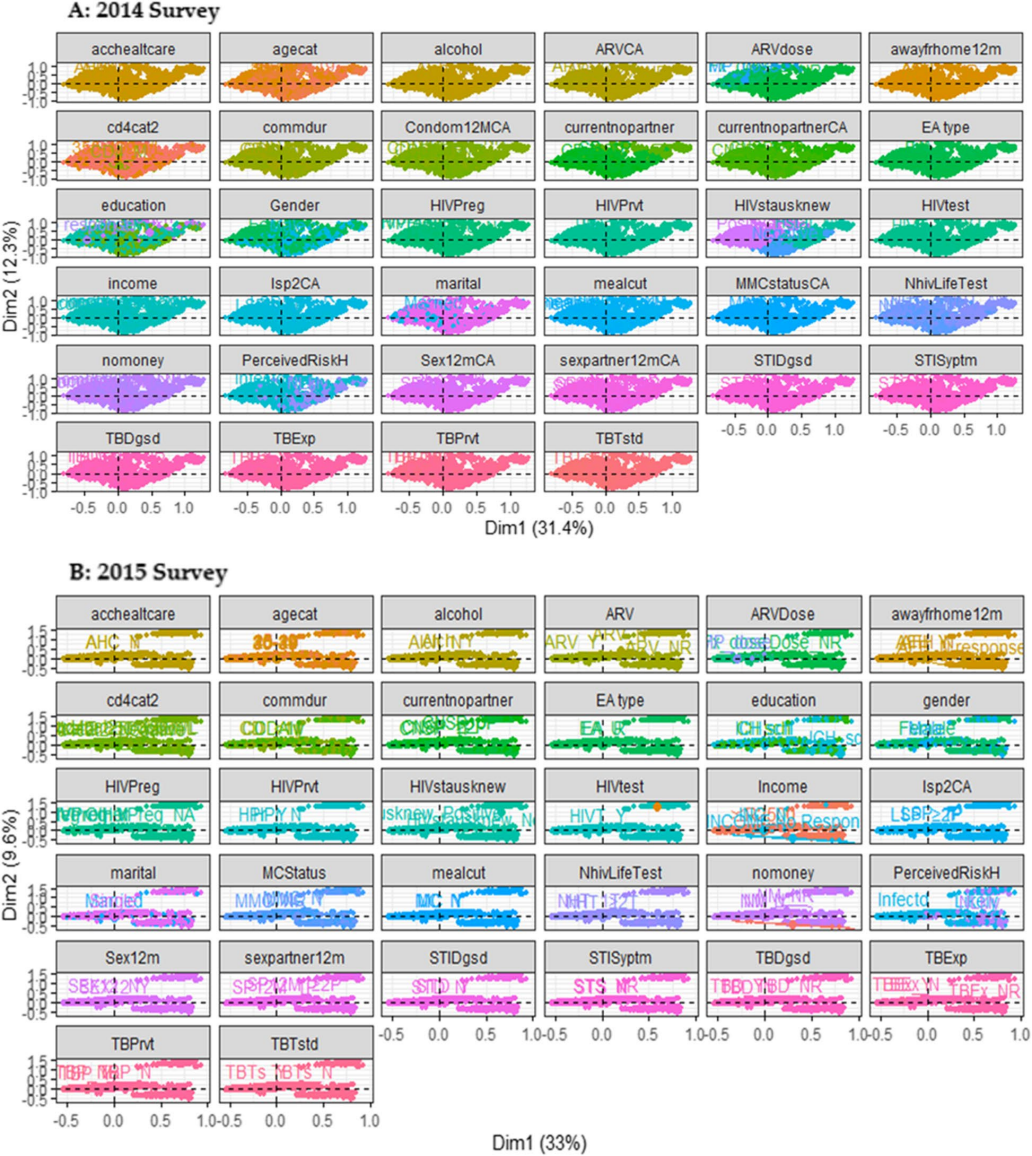
Multiple correspondence analysis factor map of individual and variables categories with 95% confidence ellipses in 2014 and 2015 survey. Factors map further show a stronger level of correlation and interaction between variables, categories and individual HIV positive men and women. In 2014 survey, factors such as perceived risk of contracting HIV, ever tested for HIV, on medication to prevent HIV, no money, had STI symptoms and ever tested for TB, while in the 2015 survey variables such as are education level, gender, ever diagnosed of TB, on medication to prevent HIV, the number of a lifetime sex partner, meal cut, on medication to prevent TB, had STI symptoms, the number of sex partner in last 12 months shows no convergence of confidence ellipses, implying these factors contribute to high viral load in this population. Also, a more clustering is observed in 2014 as compared to 2015

Furthermore, to corroborate whether the groupings of nominal indicators deviate meaningfully, we reported a 95% confidence ellipse for individual contributing factors of HHVL, as displayed in Fig. 4. The confidence ellipse gave a healthy stage of ambiguity correlation with the theme setting. In 2014 from Fig. 4a, it was observed that all levels of variables such as condom use in the last 12 months and on medication to prevent HIV, no money, ever had sexually transmitted infection (STI) symptoms and ever tested for TB in addition to the perceived risk of contracting HIV and ever tested for HIV, shows no convergence of confidence ellipses. Similarly, in the 2015 survey (Fig. 4b), all variables are significantly different from each other except for education level, gender, ever diagnosed of TB, on medication to prevent HIV, the number of lifetime sex partners, medical circumcision, meal cut, on medication to prevent TB, had STI symptoms, the number of sex partner in last 12 months shows no merging of confidence ellipses, suggesting that these indicators are central predictors of HHVL. Besides, we have observed that various potential contributing factors differ significantly based on the overlapping of 95% confidence ellipses. So, we can establish that they have contributed to high HIV RNA viral loads. This plot also reveals the similarities between each variable which aid to further test for multicollinearity before further analysis.

### Random forest analysis results

The performance assessment of the training dataset shows an overall error rate of 20.7% (21.1%) given an accuracy of 79.3% (78.9%) for 2014(2015) surveys respectively. Higher accuracy rate suggests that all the covariates are reliable for prediction across the study years.

**Fig. 5:**
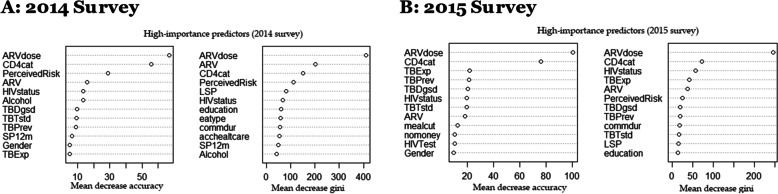
Random Forest plot showing high importance predictors of high viral load (2014 and 2015 survey). High importance predictors in 2014 are: ARV dosage, CD4 cells per μL, perceived risk of contracting HIV, ARV, knowledge of HIV status, alcohol, ever diagnosed with TB, ever tested with TB, on TB medication, total number of sex partners last 12 months, gender, total number of lifetime sex partners, place of resident, length of stay in community and education status. While in 2015 high importance predictors are: ARV dosage, CD4 cells per μL, exposed to TB last 12 months, ever diagnosed with TB, on TB medication, knowledge of HIV status, ARV, meal cut, no money, gender, perceived risk of contracting HIV, length of stay in community, total number of lifetime sex partners and education status

Random forest analysis plot gives the MDA and MDG measure of the relatively high important predictors of HHVL as shown in Table 4 and Fig. 5. In 2014 survey high important predictors are ARV dosage, CD4 cells per μL, perceived risk of contracting HIV, ARV, knowledge of HIV status, alcohol, ever diagnosed with TB, ever tested with TB, on TB medication, total number of sex partners last 12 months, gender, total number of lifetime sex partners, place of resident, education, length of stay in community and education status with their corresponding MDA and MDG: 66.9, 55.8, 28.8, 15.7, 13.5, 13.3, 9.5. 9.0, 8.9, 6.3, 4.9, 4.7, 3.9, 2.2, 1.1 and 410.8, 149.9, 111.9, 202.3, 66.8, 41.3, 35.3, 25.5, 36.2, 49.6, 39.2, 80.9, 57.4, 61.5, 56.3 were top predictors of HHVL by standardized importance. Similarly, in the 2015 survey, ARV dosage, CD4 cells per μL, exposed to TB last 12 months, ever diagnosed with TB, on TB medication, knowledge of HIV status, ARV, meal cut, no money, gender, perceived risk of contracting HIV, length of stay in community, total number of lifetime sex partners and education status with their corresponding MDA and MDG: 100.4, 76.1, 21.5, 21.1, 9.7, 18.0, 12.0, 10.5, 9.5, 1.1, 6.6, 0.8, 1.6 and 246.8, 72.5, 45.5, 42.5, 19.5, 57.9, 38.4, 13.2, 12.7, 25.1, 18.9, 15.5, 14.7 were top predictors of HHVL by standardized importance. Higher value of MDA or MDG implies the most important predictors in the model. However, other predictors shows a medium to low level of prediction, associated MDA and MDG of all predictors of High viral load are shown in Table S4 of Additional file 1.

**Table 4:** Associated Mean Decreases Accuracy (MDA) and Mean Decrease Gini (MDG) of high importance predictors of High viral load (2014–2015)

Predictors (2014 Survey)	MDA	MDG	Predictors (2015 Survey)	MDA	MDG
Dose ARV	66.9	410.8	ARV dosage	100.4	246.8
CD4 cell count	55.8	149.9	CD4 cell count	76.1	72.5
Perceived risk of contracting HIV	28.9	111.9	Exposed to TB last 12 months	21.5	45.5
On ARV	15.7	202.3	On medication to prevent TB	21.1	42.5
Knowledge of HIV status	13.5	66.8	Ever diagnosed of TB	20.2	19.5
Alcohol	13.3	41.3	Knowledge of HIV status	19.7	57.9
Ever diagnosed of TB	9.5	35.3	On ARV	18.0	38.4
Ever tested for TB	9.0	25.5	Perceived risk of contracting HIV	17.1	35.5
On TB medication	8.19	36.2	Meal cut	12.0	33.2
Total number of sex partners last 12 months	6.3	49.6	No money	10.5	33.3
Gender	4.9	39.2	Gender	9.5	12.7
Exposed to TB last 12 months	4.9	33.0	Length in community	6.6	18.9
Total number of lifetime sex partners	4.7	20.9	Ever tested for TB	9.6	16.7
Education status	1.1	21.5	Total number of lifetime sex partners	1.8	15.5
Length in community	2.2	26.3	Education status	1.6	14.7

## Discussion

Among all HIV positive men and women in this study across both surveys, LHVL of 49.9% and 59.0% was observed in year 2014 and 2015 respectively, which is sustainably below the UNAIDS targets of 86% to end the epidemic by 2030 [1, 3], and of the country with 85.7% at viral suppression threshold of < 400 copies/ml at the of 2020 [51]. However, an improvement by 9.1% was observed over the years. This reflects South Africa’s commitment and efforts in ART scale up, HIV intervention and prevention programmes towards ending the HIV epidemic by 2030 [2, 3]. The implication of this difference between the UNAIDS 1000 copies/ml and our study 400 copies/ml cut off for viral suppression is the challenge of adherence to ART in South Africa, despite having the largest ART programme globally. Likewise, treatment experts have advocated the use of lowest possible viral load threshold as the goal of HIV treatment, those in the interest of public health impact has supported the use of 1000 copies/ml threshold as a pragmatic choice [52]. However, the latter is offered as a compromise between what is ideal for individual and the need to focus on attaining the targets. Study from Botswana showed that despite achieving 90-90-90 targets with 1000 copies/ml cut off, HIV incidence rate did not change [53]. Similarly previous studies from this data shows that this target has not been met with 1000 copies/ml threshold, with increasing HIV incidence [12, 54].

The poverty level in this hyperendemic community is high, with over 50% of participants having an income index less than or equal to R2500 a month. Most participants (63.9%; 83.3%) had more than two sexual partners in their lifetime. Apart from clinical variables, socio-demographic and behavioral characteristics influenced HHVL in this area.

We identified contributing factors of HHVL amongst HIV-positive men and women, using MCA and RFA plot techniques as a data mining approach. Overall, we found a total of twenty (20) variables to be contributing factors to high viral load amongst HIV positive men and women, out of the thirty (30) variables included in the analysis, with ten (10) variables consistently identified from both methods as most identified factors. Socio-demographic factors (which include age, gender, meal cut, income loss, community duration, history of migration, accessing healthcare, behavior factors which include number of sex partners in the last 12 months, current number of sex partners, TB/STI history include exposed to TB in the last 12 months, HIV risk perception and knowledge factors include ARV use and ARV dosage, perceived risk of contracting HIV, self-reported HIV status, number of lifetime HIV test, pregnant while HIV positive and condom use last 12 months were identified contributing factors for HHVL high. These were similar to past findings in the literature [6–8, 55]. However, care should be given to generalizing these factors on both genders; separate analysis is recommended for the male and female because the biological and social circumstances with the transmission of HIV differ by sex.

Consistently most contributing factors from both methods (MCA and RFA) across both years was found to be knowledge of HIV status, being on ART, ART dosage, current CD4 cell count, perceived risk of contracting HIV, knowledge of HIV status, alcohol, total number of sex partners in the last 12 months, total number of lifetime sex partner, ever diagnosed of TB and exposure to TB in last 12 months. These were similar to past studies [5, 6, 56, 57]. In the HIV treatment cascade and especially in achieving the UNAIDS targets, knowledge of HIV-positive status is a critical entry point to HIV care. Similarly, at the individual level, ART has been shown to have considerably benefit, having transformed HIV disease, which was once an inevitably fatal disease into a chronic, manageable condition, improved life expectancy, and reduced HIV incidence [51, 57–59]. The first-line ART regimen had been simplified and improved to a single tablet of fixed-dosed combination (consisting of tenofovir, emtricitabine and efavirenz) to improve adherence. Also, PLHIV with TB comorbidity may experience virological failure. The risk of virological non-suppression may also be increased by concurrent ART and TB treatment, majorly due to impaired treatment adherence and pharmacokinetic drug interaction. Thus, PLHIV and ART with active TB should be prioritized for viral load monitoring and adherence support interventions.

Using the map distance points, the MCA plot reveals that closer variables are more related, and the farther a variable is from the center, the lesser its contribution to the eigenvalue of the respective dimension. Potential factors are colored with adjacent lines showing each variable distribution. For instance, our study reveals that factors contributing most to the first two dimensions are knowledge of HIV status and being on ARV because they are farthest from the center of the map. This is similar to both surveys, and further revealed the strength of these variables as significant potential factors contributing to high viral load in this community.

MCA and RFA were used to visualize potential predictors variables. Epidemiology, public health, social science, and behavioral studies are often faced with challenges of many responses to questions with nominal answered scales, resulting in several categorical variables in the study to measure and contribute to input in a model result multicollinearity those variables. An initial visualization and examination of the associations amongst these categorical variables would provide more accurate insight and further help identify and visualize strongly related variables before bivariate or multivariate analysis, which do not perform such standardization and visualization [24, 44].

In comparison with RFA, MCA’s strength was shown in its rich graphical and visual illustration and display of association among the explanatory categorical variables considered in this study. The findings of the study capture more information on various patterns which contingency tables do not capture. MCA has been applied to nominal, ordinal, or binary variables and complex surveys [25, 26, 30–32], often seen in survey designs as the HIPSS study design considered here. Another significant advantage of MCA is the power to mathematically break down the value of goodness of fit statistics into components due to rows and columns of the contingency Table [42]. Also, MCA makes no distributional assumptions [38], unlike conventional statistical methods, which require an underlying assumption of normality. The method of MCA further helps to reduce dimensionality with the least possible loss of information. The MCA technique helped explore how underlying socio-demographic, behavioral, Psycho-social, HIV testing history, biological and geographic variables were associated. With such a large data set, where small associations are more likely to achieve statistical significance, MCA provides a robust and meaningful analyses that account for the interaction between variables in the data set as a whole [44]. As highlighted in this study, MCA’s strengths include model-free assumption, making this method adaptable for any sizeable categorical data set. Another MCA’s strength in showing how explanatory categories from two or more variables are clustered was revealed. Further direction of combined effect and interaction of some variables was revealed. Lastly, MCA’s strength of the Greenacre adjustment method was shown in this paper, which past studies [3, 25, 26, 28–32] did not utilize. Similarly, RFA as a machine learning tool has been used in many studies with substantial success [34, 35, 48, 49], but still has limitations of no directional or pattern effects as it only predicts specific indicators contingent on the relevance of their contribution [49]. In general, key strength of our study is the robustness of the study design, high participation rates and biological measurement in a real time setting.

However, our study has some limitations; firstly, since this was a cross-sectional and a population-based study, rather than a randomized clinical trial, the duration on treatment was not applicable and more data on ART was not available. Furthermore, time to viral suppression among those initiating ARVs varied across PLHIV; we, therefore, emphasized the need for ongoing population-level surveillance to monitor individuals contributing to achieving low viral load. Although, we were not justified in undertaking a randomized controlled trial, instead, we aimed to determine whether health sector programs reached the population and measured the impact on several HIV outcome measures.

Secondly, MCA and RFA were practical exploratory techniques in identifying potential and contributing factors without causal inference; however, these methods pose some limitations and gaps upon which other advanced confirmatory statistical methods can be built. MCA is a strong technique to identify associated variables and detect patterns in large datasets. But it does not formally prove associations between measured variables and outcomes. On the other hand, RFA outputs a ranking of the relative importance of variables in classifying outcomes but does not quantify the absolute contribution of each variable in determining the outcome. Care should be taken in the choice of data exploratory approach; a multivariate graphical technique is therefore recommended because it provide more subjective analysis. A confirmatory statistical method such as structural equation modelling could be explored to assess similar outcomes.

Lastly, our results are limited to the study area and not necessarily generalizable among communities with considerable epidemic, however findings may be applicable to many other African countries where coverage of HIV programs is limited. Therefore, our analysis from MCA and RFA contributes to selection and identifying potential variables to include in a model amidst multiple variables without being bias.

## Conclusion

While the proportion of PLHIV with LHVL increased by 9.1% from 2014 to 2015, almost half 50.1% (41.0%) of HIV positive men and women had HHVL in 2014 (2015) respectively. This highlights a crucial gap in ART initiation and adherence to achieve low HIV viral load and therefore underscores the public health implication of sustained HIV transmission risk. Similarly, the MCA scheme affirmed the significant relations of alliance among socio-demographic indicators, sexual behaviour, HIV testing and history, STI and TB history and clinical factors, and characteristics of HHVL. Various distinct shapes were gotten to assist healthcare providers in the management of PLHIV proficiently. Therefore, the relations of alliance detected between socio-demographic, sexual behaviour, clinical, HIV testing and knowledge, and TB and STI history could assist in explaining precise clinical protocols for an individual pattern of HHVL. Equally, the profiles reveal assemblages of people who perhaps share the same risk factors and may afterward be targeted in health promotion and prevention policies.

Finally, our findings affirm the superiority of MCA due to its visualisation, reliability, and strength to discover connotations between categorical indicators as connected to HHVL at various individual and community levels. When faced with complex survey data and challenges of variables selection in research, exploratory data analysis with robust graphical visualisation and reliability that can reveal divers’ structure should be considered.

## Supplementary Information

**Additional file 1.**

**Additional file 2.**

**Additional file 3.**

## Data Availability

The datasets generated and analysed during the current study are available on reasonable request from principal investigator and the corresponding author. However, restrictions apply to these data’s availability due to maintaining participants’ confidentiality and the colmmunity involved.

## Abbreviation

AIDS: Acquired Immunodeficiency Syndrome
ART: Anti-retroviral Therapy
ARV: Antiretroviral drug
CAPRISA: Centre for the AIDS Programme of Research in South Africa
CD4: Cluster of differentiation 4
FA: Factor Analysis
HIPSS: HIV Incidence Provincial Surveillance System
HHVL: High HIV RNA Viral Load
HIV: Human Immunodeficiency Virus
KZN: KwaZulu-Natal
LHVL: Low HIV RNA Viral Load
MCA: Multiple Correspondence Analysis
MS: Mean Score
MDA: Mean Decreases Accuracy
MDG: Mean Decreases Gini
PCA: Principal Components Analysis
PLHIV: People living with HIV
RFA: Random Forest Analysis
SABSSM: South African National HIV Prevalence, Incidence, Behaviour and Communication Survey
STI: Sexually Transmitted Infections
UNAIDS: The Joint United Nations Programme on HIV/AIDS
WHO: World Health Organization
