# Supplementary material for: Identifying Potential Factors Associated with High HIV viral load in KwaZulu-Natal, South Africa using Multiple Correspondence Analysis and Random Forest Analysis
Source: BMC Med Res Methodol. 2022 Jun 17;22:174. doi: 10.1186/s12874-022-01625-6 (PMC9206247; doi:10.1186/s12874-022-01625-6)
Supplement: Supplementary file 1 — Additional file 1. [file 12874_2022_1625_MOESM1_ESM.docx]

**ADDITIONAL FILE 1**

**SUPPLEMENTARY TABLES**

| **Table S1: Characteristics of HIV positive participants in the 2014 and 2015 survey** | | |
| --- | --- | --- |
| **Characteristics** | **2014 Survey**  **N= 3956** | **2015 Survey**  **N=3868** |
|  | **n(%)** | **n(%)** |
| **HIV viral load** |  |  |
| ≥400 copies per (Unsuppressed HIV viral) load | 1981 (50.1) | 1584 (41.0) |
| <400 copies m/L (Suppressed HIV viral load) | 1975 (49.9) | 2284 (59.0) |
| **Socio-demographic characteristics** |  |  |
| **Gender** |  |  |
| Female | 2946 (74.5 | 2947 (76,2) |
| Male | 1010 (25.5) | 921 (23.8) |
| **Age group in years** |  |  |
| 15-19 | 166 (4.2) | 171 (4.4) |
| 20-24 | 520 (13.1) | 409 (10.6) |
| 25-29 | 748 (18.9) | 736 (19.0) |
| 30-34 | 776 (19.6) | 893 (23.1) |
| 35-39 | 721 (18.23) | 694 (17.9) |
| 40-44 | 606 (15.3) | 605 (15.6) |
| 45-49 | 419 (10.6) | 360 (9.3) |
| **Education level ^a^** |  |  |
| Complete High School | 1666 (42.1) | 1565 (40.4) |
| Incomplete High School | 2104 (53.2) | 2301 (59.5) |
| No schooling | 185 (4.7) | 0 |
| **Relationship status** |  |  |
| Ever Married | 613 (15.5) | 732 (18.9) |
| Never Married | 3343 (84.5) | 3136 (81.1) |
| **Community duration** |  |  |
| Always | 3055 (77.2) | 2023 (52.3) |
| Less than 1 year ago | 110 (2.8) | 156 (4.0) |
| More than 1 year ago | 791 (20.0) | 1689 (43.7) |
| **Income index** |  |  |
| ≤ ZAR 2500 | 2173 (54.9) | 2136 (55.2) |
| > ZAR 2500 | 949 (24.4) | 1617 (41.8) |
| No income | 525 (13.3) | 111 (2.9) |
| **Accessing healthcare** |  |  |
| Yes | 1563 (35.6) | 2826 (73.1) |
| No | 2393 (60.4) | 1042 (26.9) |
| **Meal cut** |  |  |
| Yes | 879 (22.2) | 1733 (44.8) |
| No | 3077 (77.8) | 2135 (55.2) |
| **Income loss** |  |  |
| Yes | 882 (22.3) | 1924 (49.7) |
| No | 2973 (75.1) | 1920 (49.6) |
| No response | 101 (2.6) | 24 (0.7) |
| **Migration history (away from home for >1month) ^b^** |  |  |
| Yes | 424 (10.7) | 290 (7.5) |
| No | 3519 (89.0 | 3573 (92.4) |
| **Enumeration area** |  |  |
| Rural | 1356 (34.3) | 1246 (32.2) |
| Peri-Urban | 2600 (65.7) | 2622 (67.8) |
| **Behavioural characteristics** |  |  |
| **Sex in the last 12 months** |  |  |
| Yes | 3009 (76.1) | 3387 (87.6) |
| No | 947 (23.9) | 481 (12.4) |
| **Number of sex partner last 12 months** |  |  |
| 0-1 Partners | 2479 (62.7) | 3444 (89.0) |
| 2 or more partners | 1477 (37.3) | 424 (11.0) |
| **Number of current sexual partners** |  |  |
| 0 partner | 265 (6.7) | 90 (2.3) |
| 1 partner | 3014 (76.2) | 2754 (71.2) |
| 2 or more Partners | 677 (17.1) | 1024 (26.5) |
| **Number of lifetime sexual partners** |  |  |
| 1 partner | 625 (15.8) | 645 (16.7) |
| 2 or more partners | 3331 (84.2) | 3223 (83.3) |
| **Ever consumed alcohol** |  |  |
| Never | 3086 (78.0) | 2810 (72.6) |
| Yes | 870 (22.0) | 1058 (27.4) |
| **HIV testing knowledge and history** |  |  |
| **Ever had HIV test** |  |  |
| Yes | 3257 (82.3) | 3701 (95.7) |
| No | 699 (17.7) | 467 (4.3) |
| **Number of lifetime HIV test** |  |  |
| 1 time | 1045 (26.4) | 1051 (27.2) |
| 2 or more times | 2212 (55.9) | 2650 (68.5) |
| Never | 699 (17.7) | 167 (4.3) |
| **Perceived risk of contracting HIV** |  |  |
| Already infected | 2155 (54.5) | 2622 (67.8) |
| Likely to Acquire HIV | 807 (20.4) | 669 (17.3) |
| Not likely to Acquire HIV | 994 (25.1) | 577 (14.92) |
| **STI and TB history** |  |  |
| **Ever diagnosed of STI** |  |  |
| Yes | 312 (7.9) | 431 (11.1) |
| No | 3644 (92.1) | 3437 (88.9) |
| **Ever had any STI symptoms** |  |  |
| Yes | 192 (4.9) | 114 (3.0) |
| No | 3452 (87.2) | 3323 (85.9) |
| No response | 312 (7.9) | 431 (11.1) |
| **Ever been diagnosed of TB** |  |  |
| Yes | 400 (10.1) | 559 (14.5) |
| No | 1264 (32.0) | 1639 (42.4) |
| No response | 2292(57.9) | 1670 (43.1) |
| **Exposed to TB in the last 12 months** |  |  |
| Yes | 158 (4.0) | 218 (5.6) |
| No | 2929 (74.0) | 2694 (69.7) |
| No response | 869 (22.0) | 956(24.7) |
| **Ever tested for TB** |  |  |
| Yes | 1664 (42.1) | 2198 (56.8) |
| No | 2292 (57.9) | 1670 (43.2) |
| **On medication to prevent TB** |  |  |
| Yes | 274 (6.9) | 534 (13.8) |
| No | 3282 (83.0) | 2775 (71.7) |
| No response | 400 (10.1) | 559 (14.5) |
| **On medication to prevent HIV** |  |  |
| Yes | 64 (1.6) | 126 (3.3) |
| No | 3892 (98.4) | 3742 (96.7) |
| **Clinical characteristics** |  |  |
| **ARV Dosage** |  |  |
| Fixed/single dose | 1368 (79.7) | 1966 (88.4) |
| Multiple dose | 347 (20.2) | 259 (11.6) |
| **Current CD4 cell count ^c^** |  |  |
| < 350 cells per µL | 1131 (28.6) | 966 (25.0) |
| 350 – 499 cells per µL | 880 (22.2) | 817 (21.1) |
| ≥ 500 cells per µL | 1918 (48.5) | 2072 (53.6) |
| Information missing for 1**^a^** and 2 **^a^** ; 13**^b^** and 5**^b^** ; 27**^c^** and 8**^c^** participants for 2014 and 2015 survey respectively. ZAR=South African Rand. *ZAR15~US$1; Any STI symptoms included any symptoms of abnormal vaginal discharge, burning or pain when passing urine, or presence of any genital ulcers/warts  Antiretroviral therapy (ART), Prevalence rate ratio (PRR), Confidence Interval (CI); ^a^= virally suppressed defined as HIV RNA viral load <400 copies/ml; ^c=^Includes current pregnancy | | |

| **Table S2:  Progress towards UNAIDS 95-95-95 targets among HIV positive men and women in rural KwaZulu-Natal, South Africa** | | | | | | | | |
| --- | --- | --- | --- | --- | --- | --- | --- | --- |
| **Years** | **“First 95”** | | **“Second 95”** | | **“Third 95”** |  | **Composite viral suppression€** | |
|  | **Knowledge of HIV status** | | **On ART** | | **Viral suppression^Ɏ^** | |  | |
|  | **n/N** | **% (95% CI)** | **n/N** | **% (95% CI)** | **n/N** | **% (95% CI)** | **n/N** | **% (95% CI)** |
| 2014 | 2381/3956 | 60.9 (58.4 - 63.4) | 1717/2381 | 74.3 (71.8 - 76.8) | 1393/1717 | 81.9 (79.3 - 84.4) | 1975/3956 | 49.9 (47.3 -52.6) |
| 2015 | 2810/3868 | 71.6 (69.9 - 73.4) | 2220/2810 | 78.9 (77.1 - 80.7) | 1929/2220 | 87.4 (85.8 - 89.0) | 2284/3868 | 59.0 (56.0 - 60.0) |
| **^Ɏ^**: Viral suppression among those reporting to be on ART at viral load <400 copies per mL    **^€^**: Composite viral suppression among all HIV positive at viral load <400 copies per mL | | | | | | | | |

| **Table S3: All variables and categories with their associated MCA recode in the dataset** | | | | | |
| --- | --- | --- | --- | --- | --- |
|  | | **Variables** | **MCA variable recoded** | **Categories** | **MCA categories recoded** |
|  | **Socio demographic variables** | | | | |
|  | | Gender  Age category  Education level  Relationship status  Community duration  Enumeration area  Migration history  Income index  Income loss  Accessing health care  Meal cut | **gender**  **agecat**  **education**  **marital**  **commdur**  **EA type**  **awayfrhome12m**  **income**  **nomoney**  **acchealthcare**  **mealcut** | Male  Female  15-19 ,20-24, 25-29, 30-34, 35-39, 40-44, 45-49  Complete high school  Incomplete high school  No schooling  No response  Ever married  Never married  Always  Less than 1 year ago  More than 1 year ago  Urban  Rural  Yes  No  No response  ≤R2500  >R2500  Yes  No  No response  Yes  No  No response  Yes  No  No response | **Male**  **female**  **15-19 ,20-24, 25-29, 30-34, 35-39, 40-44, 45-49**  **CH sch**  **INC sch**  **noschool**  **EDU_NR**  **Married**  **Single**  **CD_A**  **CD_<1y**  **CD_>1y**  **EA_U**  **EA_R**  **AFH_Y**  **AFH_N**  **AFH_NR**  **≤R2500**  **>R2500**  **nomoney_Y**  **nomoney_N**  **nomoney_NR**  **AHC_Y**  **AHC_N**  **ACH_NR**  **MC_Y**  **MC_N**  **MC_NR** |
|  | **Behavioural variables** | | | | |
|  | | Had sex last 12 months  Number of sex partner last 12 months  Number of current sex partner  Number of lifetime sexual partners  Condom use last 12 months  Alcohol consumption  Ever had HIV test  Number of lifetime HIV test  Perceived risk of contracting HIV  Knowledge of HIV status  On ARV | **Sex12mCA**  **Sexpartner12mCA**  **currentnopartnerCA**  **lsp2CA**  **condom12MCA**  **alcohol**  **HIVtest**  **NhivLifeTest**  **PerceivedRiskH**  **HIV status knew**  **ARVCA** | Yes  No  No response  0 partner  1 partner  2 or more partners  Refused  0 partner  1 partner  2 or more partners  1 partner  2 or more partners  Refused  Yes  No  Never  Yes  Yes  No  1 time  2 or more times  Never  Already infected  Likely to acquire HIV  Not likely to acquire HIV  Positive  Negative  Yes  No | **SEX12_Y**  **SEX12_N**  **SEX_NR**  **SP12M_0**  **SP12M_1**  **SP12M_≥2**  **SP12M_R**  **CNSP_Non**  **CNSP_1**  **CNSP_≥2**  **LSP_1**  **LSP_≥2**  **LSP_R**  **COM12_Y**  **COM12_N**  **Alch_N**  **Alch_N**  **HIVT_Y**  **HIVT_N**  **NHT_1T**  **NHT_≥2**  **NHT_Nv**  **Infectd**  **Likely**  **Nlkly**  **Positive**  **Negative**  **ARV_Y**  **ARV_N** |
|  | | **Biological/ Clinical variables** | | | |
|  | | HIV viral load  Current CD4 cell count  ARV dosage  Ever been diagnosed of STI  Had STI symptoms  Ever been diagnosed of TB  Ever tested for TB  Exposed to TB in the last 12 months  On medication to prevent TB | **Viral load profile**  **cd4cat2**  **ARVdose**  **STIDgsd**  **STISyptm**  **TBDgsd**  **TBTstd**  **TBExp**  **TBprev** | High HIV viral load  Low HIV viral load  <350 cells per µL  350-499 cells per µL  ≥ 500 cells per µL  Missing  Fixed/single dose  Multiple dose  Yes  No  Yes  No  No response  Yes  No  No response  Yes  No  Yes  No  No response  Yes  No  No response | **High viral load**  **Low viral load**    **<350**  **350-499**  **≥ 500**  **CD4_M**  **Fx_dose**  **MP_dose**  **STD_Y**  **STD_N**  **STS_Y**  **STS_N**  **STS_NR**  **TBD_Y**  **TBD_N**  **TBD_NR**  **TBT_Y**  **TBT_N**  **TBEx_Y**  **TBEx_N**  **TBEx_NR**  **TBP_Y**  **TBP_N**  **TBP_NR** |
|  | |  |  |  |  |

| **Table S4: Associated Mean Decreases Accuracy (MDA) and Mean Decrease Gini (MDG) of all predictors of High viral load (2014-2015).** | | | | | |
| --- | --- | --- | --- | --- | --- |
| **Predictors (2014 survey)** | **MDA** | **MDG** | **Predictors (2015 survey)** | **MDA** | **MDG** |
| Dose ARV | 66.9 | 410.8 | ARV dosage | 100.4 | 246.8 |
| CD4 cell count | 55.8 | 149.9 | CD4 cell count | 76.1 | 72.5 |
| Perceived risk of contracting HIV | 28.9 | 111.9 | Exposed to TB last 12 months | 21.5 | 45.5 |
| On ARV | 15.7 | 202.3 | On medication to prevent TB | 21.1 | 42.5 |
| Knowledge of HIV status | 13.5 | 66.8 | Ever diagnosed of TB | 20.2 | 19.5 |
| Alcohol | 13.3 | 41.3 | Knowledge of HIV status | 19.7 | 57.9 |
| Ever diagnosed of TB | 9.5 | 35.3 | On ARV | 18.0 | 38.4 |
| Ever tested for TB | 9.0 | 25.5 | Perceived risk of contracting HIV | 17.1 | 35.5 |
| On TB medication | 8.19 | 36.2 | Meal cut | 12.0 | 33.2 |
| Total number of sex partners last 12 months | 6.3 | 49.6 | No money | 10.5 | 33.3 |
| Gender | 4.9 | 39.2 | Gender | 9.5 | 12.7 |
| Exposed to TB last 12 months | 4.9 | 33.0 | Length in community | 6.6 | 18.9 |
| Total number of lifetime sex partners | 4.7 | 80.9 | Ever tested for TB | 9.6 | 16.7 |
| Education status | 1.1 | 61.5 | Education status | 1.6 | 14.7 |
| Length in community | 2.2 | 56.3 | Away from home last 12 months | 1.1 | 7.2 |
| Marital status | 2.7 | 35.9 | Marital status | 5.5 | 12.4 |
| Away from home last 12 months | 1.1 | 34.1 | Alcohol | 9.3 | 14.4 |
| No money | 2.6 | 36.6 | Total number of lifetime sex partners | 1.8 | 15.5 |
| Meal cut | 3.9 | 36.0 | Total number of sex partners last 2 months | 1.9 | 8.6 |
| Ever tested for HIV | 4.7 | 14.3 | Ever tested for HIV | 10.2 | 6.1 |
| Had STI symptoms | 3.4 | 30.1 | Had STI symptoms | 7.6 | 9.8 |
| Diagnosed of STI | 3.3 | 13.3 | Diagnosed of STI | 6.8 | 6.1 |
| Accessing healthcare | 1.1 | 54.1 | Accessing healthcare | 5.3 | 13.2 |
| Place of resident | 3.9 | 57.3 | Place of resident | 2.7 | 14.0 |
|  |  |  |  |  |  |

***MDA:** mean decreasing accuracy, **MDG:** Mean decreasing GINI

**Table S4: Associated Mean Decreases Accuracy (MDA) and Mean Decrease Gini (MDG) of all predictors of High viral load (2014-2015)**
