# Supplementary material for: Identifying Potential Factors Associated with High HIV viral load in KwaZulu-Natal, South Africa using Multiple Correspondence Analysis and Random Forest Analysis
Source: BMC Med Res Methodol. 2022 Jun 17;22:174. doi: 10.1186/s12874-022-01625-6 (PMC9206247; doi:10.1186/s12874-022-01625-6)
Supplement: Supplementary file 2 — Additional file 2. [file 12874_2022_1625_MOESM2_ESM.docx]

**ADDITIONAL FILE 2**

**SUPPLEMENTARY FIGURES FROM MULTIPLE CORRESPONDENCE ANALYSIS (BURT MATRIX)**


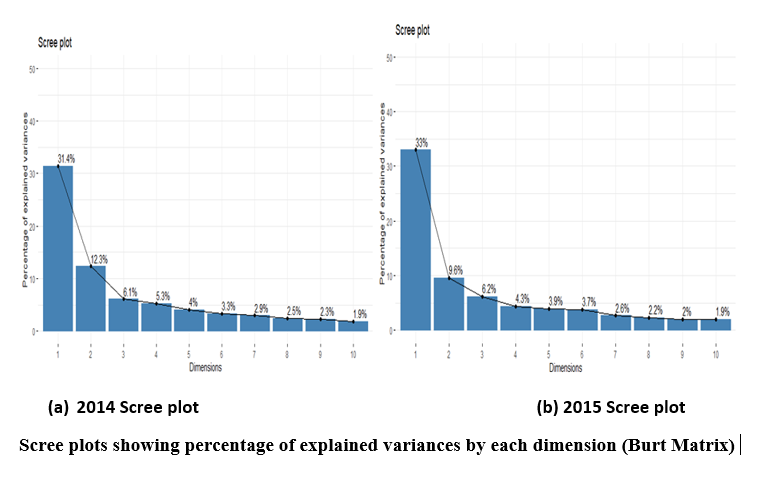


**Figure S1. Scree plot showing percentage of explained variances in top ten dimensions 2014-2015 ( Burt Matrix)**


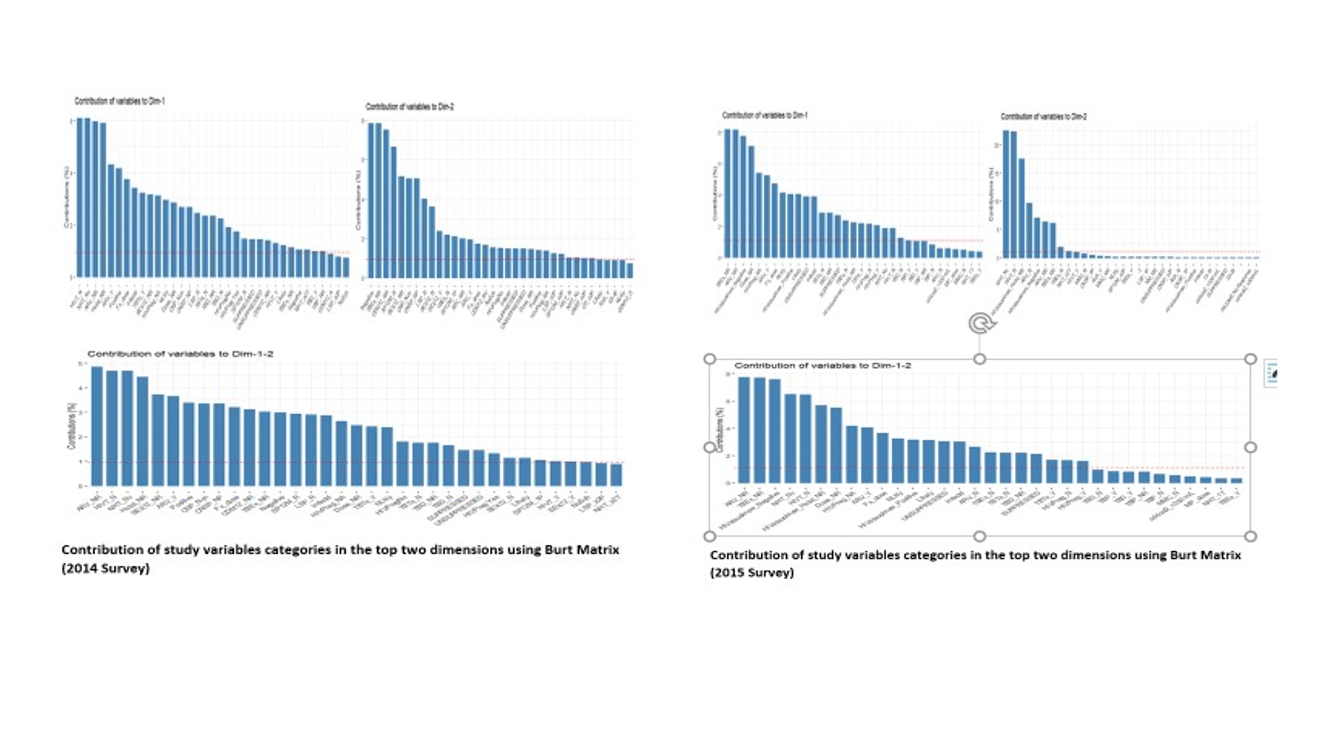


**Figure S2. Bar chart of study variables categories contribution in the top two dimensions 2014-2015 (Burt Matrix)**


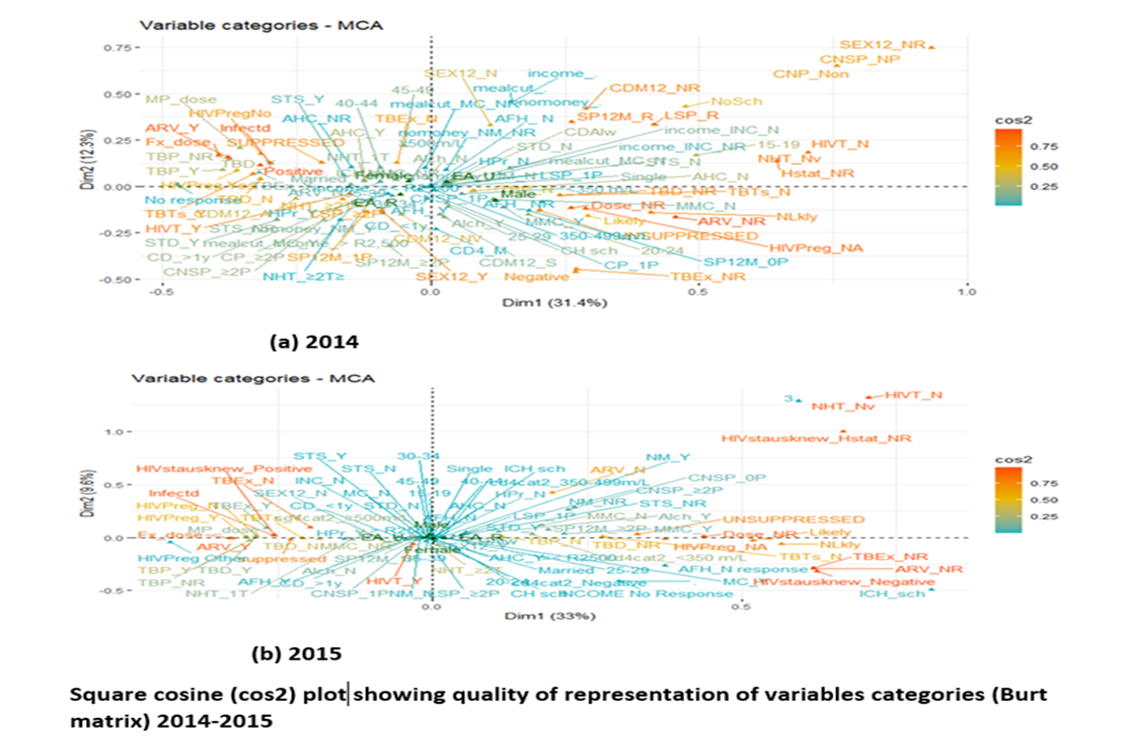


**Figure S3.** **Square cosine plot showing quality of representation of variables categories 2014-2015. (Burt matrix)**


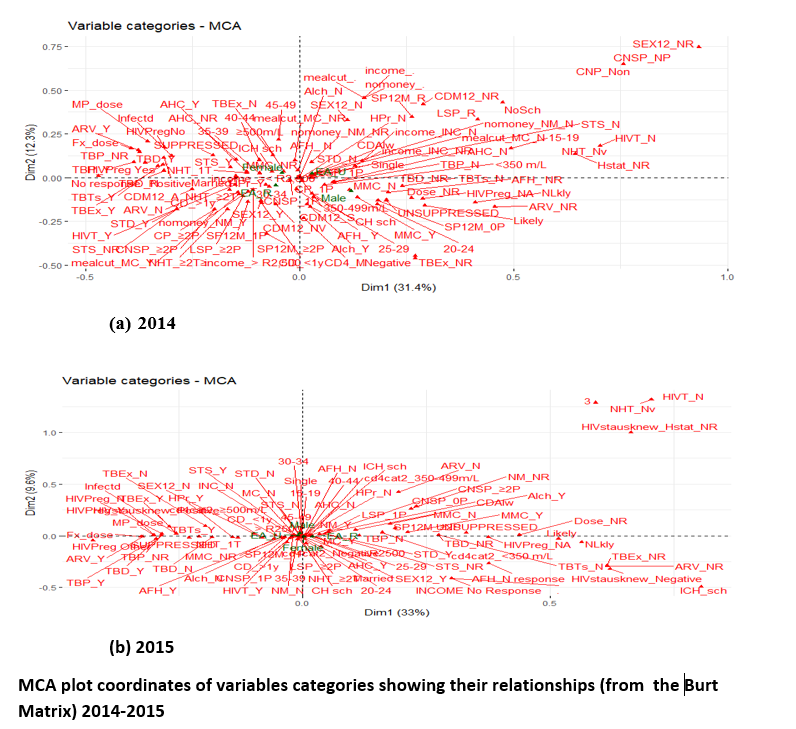


**Figure S4: MCA plot showing coordinates of study variables categories and their relative association (2014-2015) (Burt matrix)**


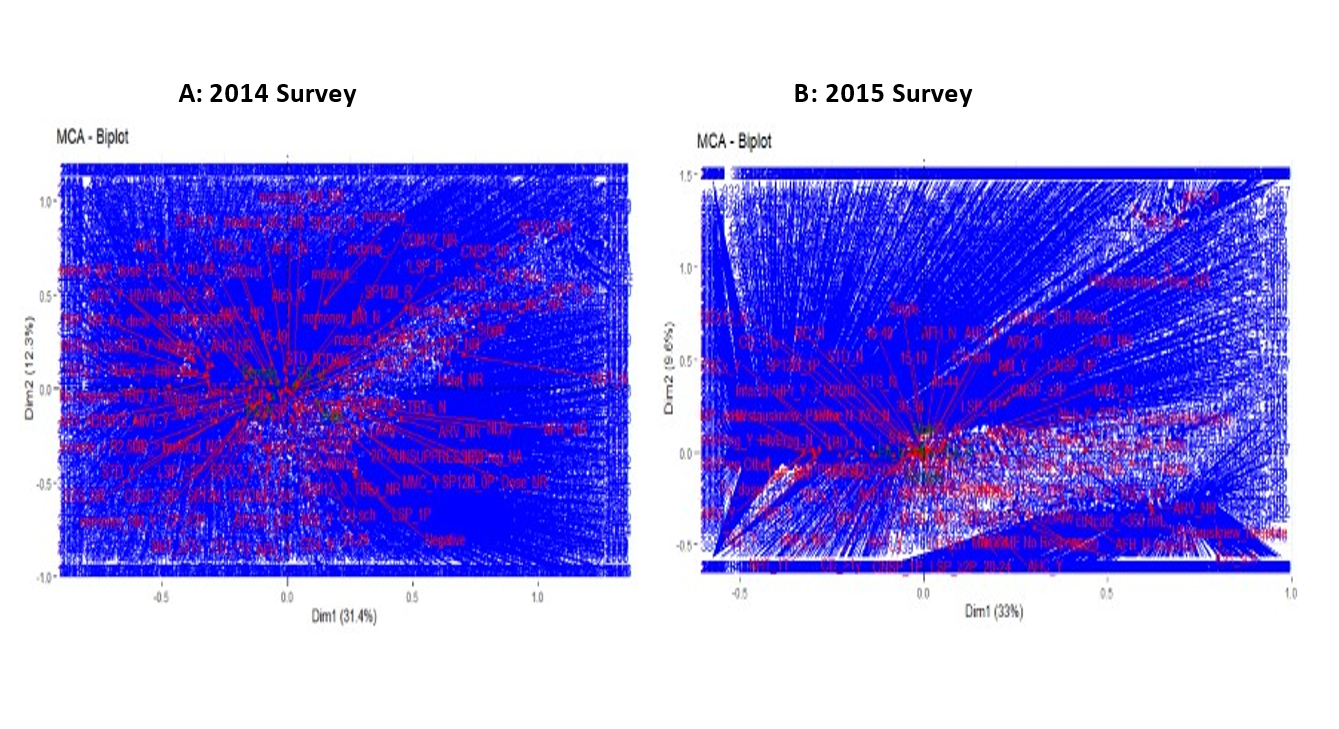


**Figure S5.** **MCA Biplot 2014-2015. (Burt matrix)**

| **Key findings**   - Multiple correspondence analysis is a multivariate technique approach used in exploring relationship within and between two or more categorical variables - Strength in the proposed method of adjustment of inertia chi-square decomposition as proposed by Greenacre (2006)   **Additional knowledge contributed**   - Multiple correspondence analysis can take several variables without restriction of the event size - Multiple correspondence analysis displays a useful, easy to read graphical display of how variables response categories are associated and interact. - Multiple correspondence analysis of binary, ordinal and nominal variables are executed without any underlying distribution assumptions. - Multiple correspondence analysis preserves the categorical nature of the variables and can allow continuous variables to be categorised.   **Way forward (what should change now)**   - Epidemiologists and most survey data analyst should see multiple correspondence analysis as their “starter tool” for data exploration and descriptive statistics. - Method of multiple correspondence analysis should include the adjustment of the dimension as this improve the variability of the data and eliminate the haste in concluding that the percentage of variation is low, especially with large complex data sets |
| --- |
