# Supplementary material for: Identifying Potential Factors Associated with High HIV viral load in KwaZulu-Natal, South Africa using Multiple Correspondence Analysis and Random Forest Analysis
Source: BMC Med Res Methodol. 2022 Jun 17;22:174. doi: 10.1186/s12874-022-01625-6 (PMC9206247; doi:10.1186/s12874-022-01625-6)
Supplement: Supplementary file 3 — Additional file 3. [file 12874_2022_1625_MOESM3_ESM.docx]

| **Table S3: All variables and categories with their associated MCA recode in the dataset** | | | | | |
| --- | --- | --- | --- | --- | --- |
|  | | **Variables** | **MCA variable recoded** | **Categories** | **MCA categories recoded** |
|  | **Socio demographic variables** | | | | |
|  | | Gender  Age category  Education level  Relationship status  Community duration  Enumeration area  Migration history  Income index  Income loss  Accessing health care  Meal cut | **gender**  **agecat**  **education**  **marital**  **commdur**  **EA type**  **awayfrhome12m**  **income**  **nomoney**  **acchealthcare**  **mealcut** | Male  Female  15-19 ,20-24, 25-29, 30-34, 35-39, 40-44, 45-49  Complete high school  Incomplete high school  No schooling  No response  Ever married  Never married  Always  Less than 1 year ago  More than 1 year ago  Urban  Rural  Yes  No  No response  ≤R2500  >R2500  Yes  No  No response  Yes  No  No response  Yes  No  No response | **Male**  **female**  **15-19 ,20-24, 25-29, 30-34, 35-39, 40-44, 45-49**  **CH sch**  **INC sch**  **noschool**  **EDU_NR**  **Married**  **Single**  **CD_A**  **CD_<1y**  **CD_>1y**  **EA_U**  **EA_R**  **AFH_Y**  **AFH_N**  **AFH_NR**  **≤R2500**  **>R2500**  **nomoney_Y**  **nomoney_N**  **nomoney_NR**  **AHC_Y**  **AHC_N**  **ACH_NR**  **MC_Y**  **MC_N**  **MC_NR** |
|  | **Behavioural variables** | | | | |
|  | | Had sex last 12 months  Number of sex partner last 12 months  Number of current sex partner  Number of lifetime sexual partners  Condom use last 12 months  Alcohol consumption  Ever had HIV test  Number of lifetime HIV test  Perceived risk of contracting HIV  Knowledge of HIV status  On ARV | **Sex12mCA**  **Sexpartner12mCA**  **currentnopartnerCA**  **lsp2CA**  **condom12MCA**  **alcohol**  **HIVtest**  **NhivLifeTest**  **PerceivedRiskH**  **HIV status knew**  **ARVCA** | Yes  No  No response  0 partner  1 partner  2 or more partners  Refused  0 partner  1 partner  2 or more partners  1 partner  2 or more partners  Refused  Yes  No  Never  Yes  Yes  No  1 time  2 or more times  Never  Already infected  Likely to acquire HIV  Not likely to acquire HIV  Positive  Negative  Yes  No | **SEX12_Y**  **SEX12_N**  **SEX_NR**  **SP12M_0**  **SP12M_1**  **SP12M_≥2**  **SP12M_R**  **CNSP_Non**  **CNSP_1**  **CNSP_≥2**  **LSP_1**  **LSP_≥2**  **LSP_R**  **COM12_Y**  **COM12_N**  **Alch_N**  **Alch_N**  **HIVT_Y**  **HIVT_N**  **NHT_1T**  **NHT_≥2**  **NHT_Nv**  **Infectd**  **Likely**  **Nlkly**  **Positive**  **Negative**  **ARV_Y**  **ARV_N** |
|  | | **Biological/ Clinical variables** | | | |
|  | | HIV viral load  Current CD4 cell count  ARV dosage  Ever been diagnosed of STI  Had STI symptoms  Ever been diagnosed of TB  Ever tested for TB  Exposed to TB in the last 12 months  On medication to prevent TB | **Viral load profile**  **cd4cat2**  **ARVdose**  **STIDgsd**  **STISyptm**  **TBDgsd**  **TBTstd**  **TBExp**  **TBprev** | High HIV viral load  Low HIV viral load  <350 cells per µL  350-499 cells per µL  ≥ 500 cells per µL  Missing  Fixed/single dose  Multiple dose  Yes  No  Yes  No  No response  Yes  No  No response  Yes  No  Yes  No  No response  Yes  No  No response | **High viral load**  **Low viral load**    **<350**  **350-499**  **≥ 500**  **CD4_M**  **Fx_dose**  **MP_dose**  **STD_Y**  **STD_N**  **STS_Y**  **STS_N**  **STS_NR**  **TBD_Y**  **TBD_N**  **TBD_NR**  **TBT_Y**  **TBT_N**  **TBEx_Y**  **TBEx_N**  **TBEx_NR**  **TBP_Y**  **TBP_N**  **TBP_NR** |
|  | |  |  |  |  |
